# Neprilysin Inhibitors in Heart Failure: The Science, Mechanism of Action, Clinical Studies, and Unanswered Questions

**DOI:** 10.1016/j.jacbts.2022.05.010

**Published:** 2022-09-07

**Authors:** Biykem Bozkurt, Ajith P. Nair, Arunima Misra, Claire Z. Scott, Jamal H. Mahar, Savitri Fedson

**Affiliations:** a Winters Center for Heart Failure Research, Cardiovascular Research Institute, Baylor College of Medicine, DeBakey Veterans Affairs Medical Center, Houston Texas, USA; b Cardiology, Department of Medicine, Baylor College of Medicine, Houston, Texas, USA; c Michael E. DeBakey Veterans Affairs Medical Center, Houston Texas, USA

**Keywords:** neprilysin, neprilysin inhibitor, NEP inhibitor, angiotensin receptor–neprilysin inhibitor, ARNi, heart failure, sacubitril, sacubitril/valsartan, Aβ, amyloid beta, ACE, angiotensin-converting enzyme, ANP, atrial natriuretic peptide, ARB, angiotensin receptor blocker, ARN, angiotensin receptor–neprilysin, BNP, brain natriuretic peptide, BP, blood pressure, cGMP, cyclic guanosine monophosphate, CSF, cerebrospinal fluid, EF, ejection fraction, eGFR, estimated glomerular filtration rate, FDA, U.S. Food and Drug Administration, GFR, glomerular filtration rate, HF, heart failure, HFpEF, heart failure with preserved ejection fraction, HFrEF, heart failure with reduced ejection fraction, LV, left ventricular, LVEF, left ventricular ejection fraction, MI, myocardial infarction, NT-proBNP, N-terminal pro–brain natriuretic peptide, NYHA, New York Heart Association, PDE, phosphodiesterase, RAAS, renin-angiotensin-aldosterone system, UACR, urinary albumin/creatine ratio

## Abstract

This article provides a contemporary review and a new perspective on the role of neprilysin inhibition in heart failure (HF) in the context of recent clinical trials and addresses potential mechanisms and unanswered questions in certain HF patient populations. Neprilysin is an endopeptidase that cleaves a variety of peptides such as natriuretic peptides, bradykinin, adrenomedullin, substance P, angiotensin I and II, and endothelin. It has a broad role in cardiovascular, renal, pulmonary, gastrointestinal, endocrine, and neurologic functions. The combined angiotensin receptor and neprilysin inhibitor (ARNi) has been developed with an intent to increase vasodilatory natriuretic peptides and prevent counterregulatory activation of the angiotensin system. ARNi therapy is very effective in reducing the risks of death and hospitalization for HF in patients with HF and New York Heart Association functional class II to III symptoms, but studies failed to show any benefits with ARNi when compared with angiotensin-converting enzyme inhibitors or angiotensin receptor blocker in patients with advanced HF with reduced ejection fraction or in patients following myocardial infarction with left ventricular dysfunction but without HF. These raise the questions about whether the enzymatic breakdown of natriuretic peptides may not be a very effective solution in advanced HF patients when there is downstream blunting of the response to natriuretic peptides or among post-myocardial infarction patients in the absence of HF when there may not be a need for increased natriuretic peptide availability. Furthermore, there is a need for additional studies to determine the long-term effects of ARNi on albuminuria, obesity, glycemic control and lipid profile, blood pressure, and cognitive function in patients with HF.

## Highlights

• Neprilysin cleaves natriuretic peptides, bradykinin, adrenomedullin, substance P, angiotensin I and II, and endothelin.
• In patients with very advanced HF, the downstream response to natriuretic peptides is blunted, and neprilysin inhibition does not appear to add benefit.
• In post-MI patients without HF, there may not be a need for increased natriuretic peptide availability with neprilysin inhibition.
• Long-term studies are needed to determine the effects of angiotensin receptor–neprilysin inhibitors on albuminuria, obesity, glycemic control, blood pressure, and cognitive function in patients with HF.

Neprilysin is a zinc-activated endopeptidase that cleaves peptides up to 40 to 50 amino acids and has a broad role in cardiovascular, renal, pulmonary, gastrointestinal, endocrine, and neurologic functions (Central Illustration). This endopeptidase was identified by unrelated investigators at different times and given different names. Neprilysin was initially described in 1973 as a neutral proteinase in rat kidney brush border membranes.^1^ A few years later, it was independently described as a brain enzyme responsible for the inactivation of enkephalin and was called enkephalinase.^2^ Subsequently, in the 1980s, it was discovered that the enzyme that was identified to break down substance P and enkephalin was identical to the endopeptidase of kidney microvilli and was given the common name of endopeptidase.^3^ Clinicians may be surprised to learn that that *common acute lymphoblastic leukemia antigen* (CALLA), an important cell surface marker for diagnosis and prognosis of acute lymphoblastic leukemia^4^; cluster of differentiation 10 (CD10), the immunohistochemical marker correlating to a higher histologic grade, larger tumor size, metastasis, and survival rate in patients with certain solid tumors^5^^,^^6^; and skin fibroblast elastase, implicated in skin aging and wrinkle formation, are also identical to neprilysin^7^ (Table 1).

**Central Illustration:**
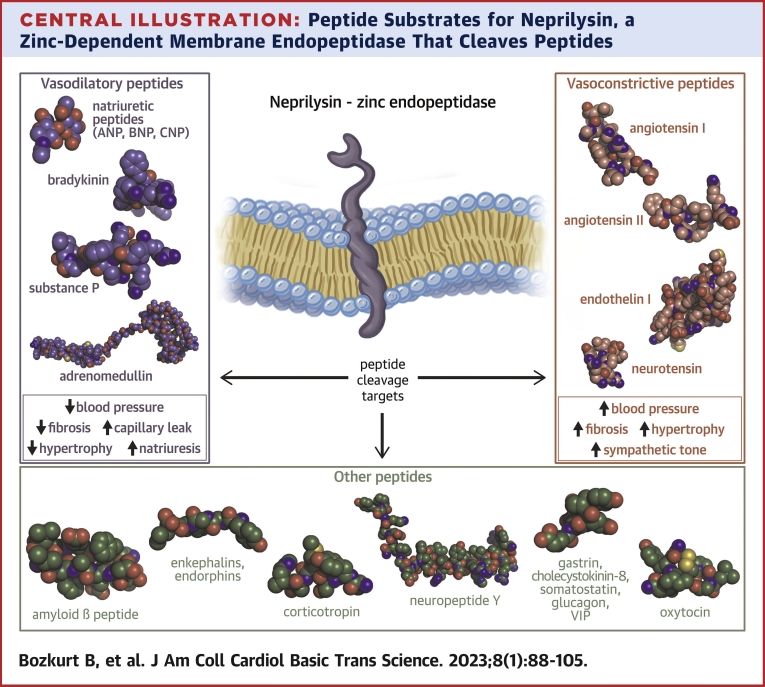
Peptide Substrates for Neprilysin, a Zinc-Dependent Membrane Endopeptidase That Cleaves Peptides Peptides that are important for cardiovascular and other systems are included. These include vasodilatory peptides (listed on the **left**) such as natriuretic peptides, bradykinin, adrenomedullin, and substance P; vasoconstrictor peptides (listed on the **right**) such as angiotensin I and II, endothelin, and neurotensin; and other peptides (listed at the **bottom**) implicated in pathways related to amyloid deposition, pain sensorium, mood, gastrointestinal processes, and metabolism such as amyloid beta peptide, enkephalins, endomorphins, corticotropin, neuropeptide Y, gastrin, cholecystokinin 8, somatostatin, glucagon, vasoactive intestinal peptide, and oxytocin.

**Table 1:** Neprilysin, an Identical Proteinase With Multiple Aliases Across Different Systems

Different Names of Neprilysin Across Different Systems
Neutral proteinase: identified in rat kidney brush border membranes
Enkephalinase: rediscovered as a brain enzyme responsible for the inactivation of enkephalin
Endopeptidase: can cleave a wide range of peptides such as substance P, given a common name
Common acute lymphoblastic leukemia antigen (CALLA): important cell surface marker for the diagnosis of acute lymphoblastic leukemia, present in 85% of cases
CD10: a marker for cancer prognosis (breast, adenocancer, others)
Skin fibroblast elastase: role in skin aging and UVA-induced skin damage, wrinkle formation

**Figure 1:**
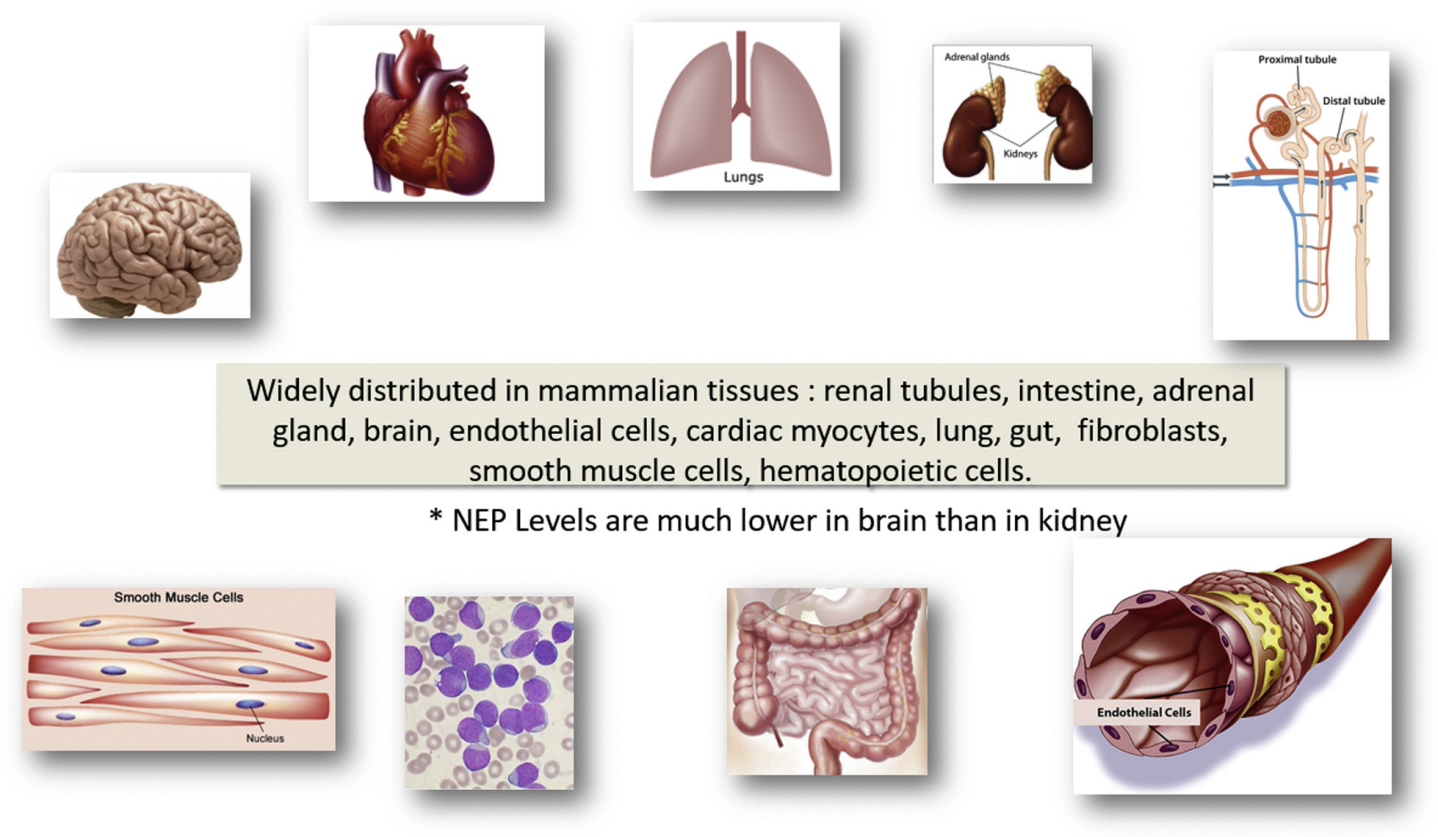
Tissue and Organ Distribution of Neprilysin Neprilysin is widely distributed in most mammalian tissues but with varying expression. For example, neprilysin levels tend to be lower in brain than in kidney, where they are mostly expressed in renal tubules. NEP = neprilysin.

Neprilysin is widely distributed in mammalian tissues, including the renal tubules, intestine, adrenal gland, brain, endothelial cells, cardiac myocytes, lung, gut, fibroblasts, smooth muscle cells, and hematopoietic cells^8^^,^^9^ (Figure 1). The highest concentrations are found in the proximal tubule of the nephrons, and its soluble form is found in the circulation, urine, and cerebrospinal fluid (CSF). Neprilysin levels are much lower in the brain than in the kidneys. Soluble neprilysin levels are elevated in patients with heart failure (HF) and are predictive of cardiovascular death and HF hospitalization in HF patients.^10^

Neprilysin has putative roles in the modulation of peptides implicated in the cardiovascular system and in other systems related to amyloid deposition, opioid receptor and pain processing, gastrointestinal processes, metabolism, sperm motility, and skin aging. There are more than 50 peptide targets of neprilysin, which include vasodilatory peptides such as natriuretic peptides, bradykinin, adrenomedullin, and substance P; vasoconstrictor peptides such as angiotensin I and II, endothelin, and neurotensin; and other peptides implicated in pathways related to amyloid deposition, pain sensorium, mood, gastrointestinal processes, and metabolism, such as amyloid beta (Aβ) peptide, enkephalins, endomorphins, corticotropin, neuropeptide Y, gastrin, cholecystokinin-8, somatostatin, glucagon, vasoactive intestinal peptide (VIP), and oxytocin, among others (Central Illustration).

## Neprilysin as a Target in Cardiovascular Disease

Although neprilysin has a broad role across different organ systems, its cardiovascular effects have resulted in paradigm-changing therapies in HF in the last decade.^11^ Exploiting the neurohormonal benefits of natriuretic peptides has been a focus in HF since the discovery of these peptides in the 1980s.^12^ Natriuretic peptides are eliminated through degradation by neprilysin and through natriuretic peptide clearance receptors.^13^ Neprilysin has a high affinity for atrial natriuretic peptide (ANP) and C-type natriuretic peptide and a lower affinity for brain natriuretic peptide (BNP).^14^^,^^15^ Natriuretic peptides cause vasodilation by stimulating particulate guanylate cyclase to produce cyclic guanosine monophosphate (cGMP). ANP and BNP promote natriuresis, diuresis, and vasodilation and have salutary effects of suppressing the renin-angiotensin-aldosterone (RAAS) axis, sympathetic nervous system, and, in turn, cardiac hypertrophy and fibrosis. There is evidence of increased enzymatic degradation of natriuretic peptides by increased neprilysin activity in HF.^16^ In animal models of severe HF, there is a significant increase in renal neprilysin activity and neprilysin messenger RNA expression, suggesting enhanced NP degradation.^17^ Cardiac neprilysin activity and messenger RNA expression are elevated in patients with HF and are related to increases in end-diastolic pressures.^16^ Thus, increased enzymatic degradation of natriuretic peptides was seen as a potential target for treatment in HF.

Neprilysin also displays enzyme promiscuity by breaking down angiotensin II and, by this mechanism, can elevate blood pressure (BP).^18^, ^19^, ^20^ In addition, neprilysin degrades bradykinin, which is a potent vasodilator, through the stimulation of endothelial nitric oxide production, is implicated in vasogenic edema, and can cause angioedema in excess.

**Figure 2:**
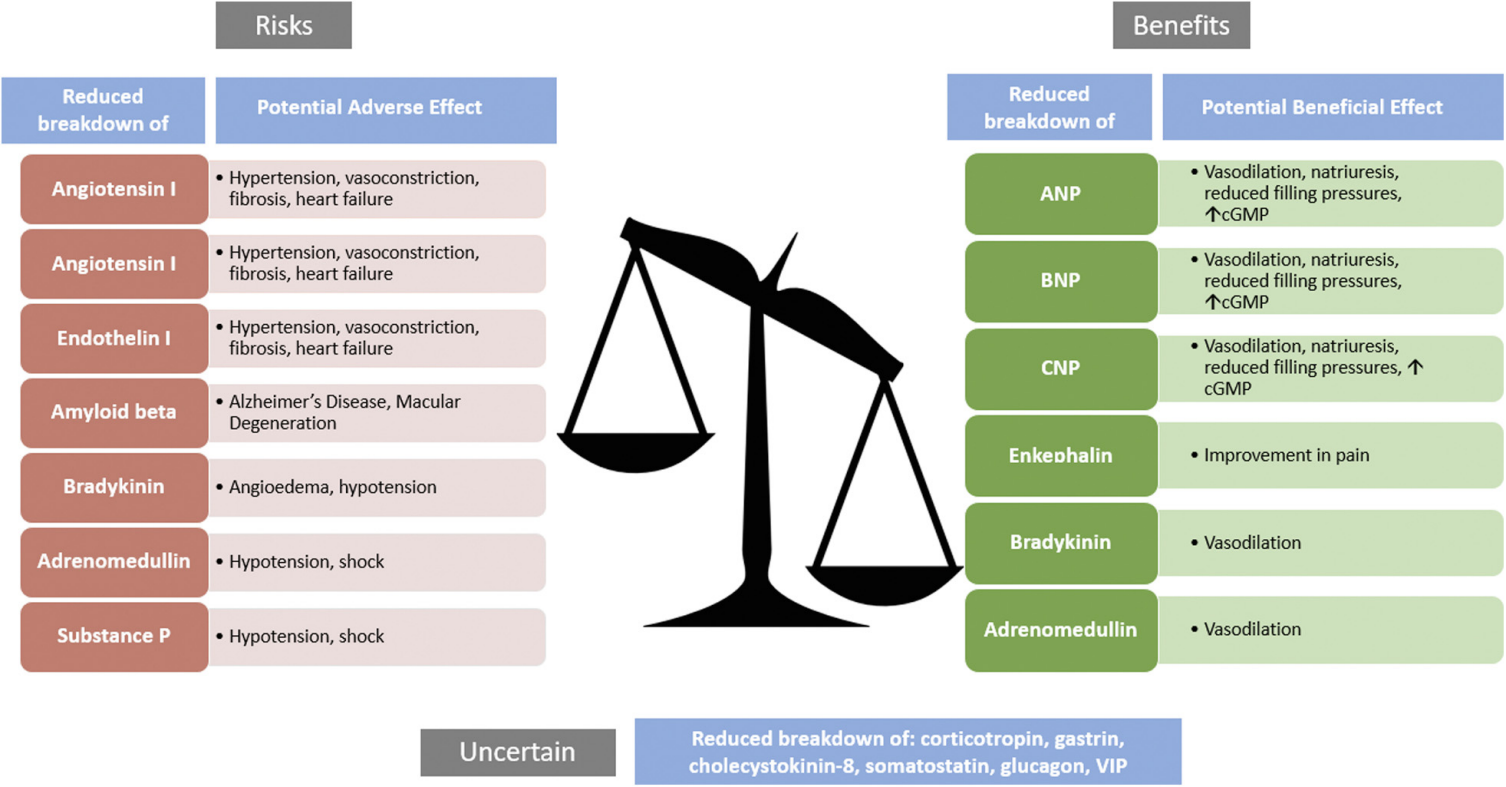
Balance of Neprilysin Inhibition Neprilysin inhibition can result in prevention of the degradation of peptides with potential beneficial effects (benefits, shown at the **right**) vs adverse effects (risks, shown at the **left**) in patients with heart failure. The significance of the reduced breakdown of certain peptides remains uncertain (shown at the **bottom**). ANP = atrial natriuretic peptide; BNP = brain natriuretic peptide; cGMP = cyclic guanosine monophosphate; CNP = C-type natriuretic peptide; VIP = vasoactive intestinal peptide.

The opposing roles of neprilysin in the degradation of both vasodilatory and vasoconstricting substrates is key to the recognition of the contrasting outcomes of neprilysin inhibition when used in isolation vs in combination with angiotensin-converting enzyme (ACE) inhibitors and angiotensin receptor blockers (ARBs). By increasing endogenous natriuretic peptide availability, neprilysin inhibition can reduce fibrosis and hypertrophy while increasing natriuresis and diuresis. However, counter to this effect is neprilysin’s role in inactivating vasoconstrictor peptides including angiotensin I, angiotensin II, endothelin I, and neurotensin. (Central Illustration, Figure 2).

## Off-Target Effects of Neprilysin, Neprilysin Deficiency, and Neprilysin Inhibition

Deficiencies in neprilysin have been associated with certain pathologic effects. Neprilysin knockout mice, although developmentally normal, have significantly lower BP and are sensitive to endotoxic shock with widespread basal plasma extravasation in post-capillary venular endothelia.^21^ These effects that are attributed to increases in substance P and bradykinin levels provide insights into BP-lowering effects with neprilysin inhibitors.

Neprilysin functions along with other peptidases to also degrade enkephalins, which are endogenous opioids that are expressed throughout the nervous system and multiple organ systems.^22^ Cardiac failure and hypertrophy can lead to activation of the cardiac opioid system, where enkephalins play a complex role in response to myocardial injury.^23^ Higher levels of proenkephalin, a stable surrogate for enkephalin, have been associated with more advanced HF, glomerular and tubular damage, and increased mortality.^24^ The questions of whether the proenkephalin levels in HF patients are markers of disease severity or are markers of maladaptive counter-regulation by an overactivated opioid system remain unanswered.^23^ In the nervous system, inhibition of enkephalin catabolism has a potential therapeutic role in the management of chronic pain disorders and mood stabilization.^25^ Treatment with neprilysin inhibitors or angiotensin receptor–neprilysin (ARN) inhibitors can result in inhibition of the degradation of enkephalin and a rise in enkephalin levels. Whether such potential increases in enkephalin levels can explain or potentiate the beneficial effects of ARN inhibitors, including improvement in symptoms and quality of life in HF patients, remain unknown.^23^

Another key neurologic role of neprilysin is in the aging brain. Alzheimer disease is marked by an abnormal accumulation of soluble and insoluble Aβ content. Neprilysin breaks down Aβ and is central to its elimination.^26^ Mice deficient for the neprilysin gene and neprilysin knockout mice have increased Aβ accumulation in the brain.^26^^,^^27^ Neprilysin inhibition increases beta amyloid in wild-type and neprilysin-deficient mice.^28^ Polymorphisms leading to loss of function in the neprilysin gene have been associated with an increased susceptibility to Alzheimer disease, especially when associated with other enzymatic deficiencies.^29^, ^30^, ^31^ Overexpression of neprilysin or neprilysin gene transfer reverses the Alzheimer phenotype in mouse models.^27^

Aβ accumulation with neprilysin inhibition has also been implicated in macular degeneration.^32^ Aβ levels were reduced in mouse eye tissues by intravitreally delivered neprilysin.^33^ Beyond the central nervous system, neprilysin has additional neuroendocrine roles. Adrenocorticotropin, which is produced and secreted by the posterior pituitary gland and stimulates cortisol release from the adrenal cortex, is cleaved by neprilysin. Oxytocin, a neuropeptide produced by the hypothalamus and secreted by the posterior pituitary gland, also undergoes hydrolysis by neprilysin. The significance of elimination of the breakdown of these peptides is unclear.

Neprilysin also plays a potential role in obesity. Neprilysin-deficient mice become obese under a normocaloric diet, characterized by deregulation of lipid metabolism and manifested by impaired glucose tolerance with higher blood glucose and triglyceride levels and lower high-density lipoprotein cholesterol levels. They demonstrate age-related obesity, with visceral fat accumulation and insulin resistance attributed to down-regulation of anorexigenic peptides influenced by neprilysin activity.^34^ These metabolic changes have not been observed in clinical trials.

Neprilysin is also implicated in skin aging, UV-induced skin damage, wrinkle formation, and neonatal development.^7^^,^^35^ Neprilysin activity is markedly enhanced in human keratinocytes and human skin fibroblasts in a pattern similar to aging and is associated with wrinkle formation and damage after exposure to UV light and irradiation exposure. Topical neprilysin inhibitors are being tested for wrinkle prevention.^7^^,^^36^

## Evidence with Neprilysin Inhibition Alone

Early experimental investigations focused on the role of neprilysin inhibition in potentiating the effects of natriuretic peptides. Based on early experimental results and the awareness of the role of natriuretic peptides in volume overload and hypertension, the selective inhibition of neutral endopeptidases underwent clinical trials. Early studies with inhibitors of neprilysin such as candoxatril, ecadotril, or acetorphan resulted in augmentation of endogenous ANP availability,^37^ promoted diuresis, and reduced right atrial pressure and pulmonary capillary wedge pressure in subjects with HF.^38^, ^39^, ^40^, ^41^, ^42^, ^43^, ^44^ Larger studies had negative results and demonstrated harm. Studies of ecadotril were halted for safety concerns because of reports of sudden cardiac death and severe drug-induced aplastic anemia. Candoxatril was associated with increases in plasma renin activity and angiotensin II and epinephrine levels,^45^, ^46^, ^47^, ^48^, ^49^, ^50^ especially at high doses.^51^ Among healthy adults, candoxatril lowered central venous pressure but increased epinephrine and endothelin 1 levels and resulted in an increase in systolic BP.^52^ Candoxatril was voluntarily withdrawn after additional studies failed to show benefit in reducing BP. The opposing effects of neprilysin in the degradation of both vasodilatory and vasoconstrictor peptides provide a potential explanation for some of the negative results of neprilysin inhibition in hypertension and chronic HF.^53^

## Studies with a Combination of Neprilysin and Ace Inhibitors

Although neprilysin inhibition increases the concentration of bradykinin, adrenomedullin, and circulating natriuretic peptides, their beneficial effects are counteracted by increases in the concentrations of angiotensin II and endothelin I. Thus, combining ACE inhibitors with neprilysin inhibitors was mechanistically reasonable.

In early studies, the combination of neprilysin inhibitors with ACE inhibitors showed increased synergistic efficacy for BP lowering in patients with hypertension.^54^ Subsequent studies showed promise with omapatrilat, a vasopeptidase inhibitor with combined neprilysin and ACE inhibition in patients with HF and hypertension.^55^ In OVERTURE (Omapatrilat vs Enalapril Randomized Trial of Utility in Reducing Events), a long-term randomized study in patients with HF with reduced ejection fraction (HFrEF) and a recent HF hospital admission, omapatrilat was noninferior but not superior to enalapril. Adverse events including HF, hypotension, and dizziness occurred similarly in both groups, with angioedema reported in 24 (0.8%) omapatrilat- and 14 (0.5%) enalapril-treated patients.^56^ In the larger OCTAVE (Omapatrilat Cardiovascular Treatment vs Enalapril) trial of 25,302 patients with untreated or uncontrolled hypertension, omapatrilat reduced systolic BP 3.6 mm Hg more than enalapril and was associated with less use of adjunctive antihypertensive therapy. Overall death rates and adverse events were similar. However, angioedema was more frequent with omapatrilat than enalapril (2.17% vs 0.68%) and was associated with airway compromise. The rates of angioedema were much higher in Black individuals (5.54% for omapatrilat and 1.62% for enalapril) and in smokers (3.93% for omapatrilat and 0.81% for enalapril).^57^ The lack of superiority of omapatrilat compared to enalapril in HF trials and the risk and severity of angioedema in hypertension trials forced the withdrawal of omapatrilat from consideration of approval by the U.S. Food and Drug Administration (FDA). The mechanism for the increased risk in angioedema is likely attributable to an increase in circulating bradykinins caused by inhibition of both ACE and neprilysin. Given that all drugs in this class potentially have a high risk of angioedema, the enthusiasm to further study the combination of neprilysin inhibitors and ACE inhibitors has dissipated. With recognition that neprilysin inhibition by itself is ineffective and that the combination of neprilysin and ACE inhibition is associated with an increased risk of angioedema, the combination of angiotensin receptor and neprilysin inhibition was proposed as a safer and more effective option because ARBs do not increase bradykinin levels and are not associated with as much angioedema risk as ACE inhibitors.

## Clinical Evidence of Angiotensin Receptor–Neprilysin Inhibitors

Over the last 10 to 15 years, several trials sought to characterize the benefits of ARN inhibitors. Sacubitril-valsartan is the specific formulation of ARN inhibitors that is available and widely used in practice. There have been several seminal trials examining the benefit of ARN inhibitors in patients with HFrEF,^11^^,^^58^^,^^59^ HF with preserved ejection fraction (HFpEF),^60^ and patients post–myocardial infarction (MI) with high-risk features for developing HF^61^ (Table 2).

**Table 2:** Clinical Studies With ARNi in Patients With Heart Failure

Study Publication Year	Patient Population	Inclusion Criteria	Intervention/Comparator	Primary Endpoint Results
PARAMOUNT^65^ 2012	HFpEF	Patients ≥40 y of age LVEF ≥45% NYHA functional class II-III HF NT-proBNP > 400 pg/mL	LCZ696 (sacubitril valsartan) vs valsartan	Significant reduction in NT-proBNP in LCZ696 group vs valsartan (ratio of change: 0.77; 95% CI: 0.64-0.92; *P =* 0.005)
PARADIGM-HF^11^ 2014	HFrEF	NYHA functional class II- IV LVEF ≤35% BNP >150 pg/mL hospitalized for HF ≤12 months	LCZ696 (sacubitril valsartan) vs enalapril	CV death and heart failure hospitalization lower in LCZ696 group vs enalapril: 914 (21.8%) vs 1,117 (26.5%), respectively (HR: 0.80; 95% CI: 0.73-0.87; *P <* 0.001)
TITRATION^62^ 2016	HFrEF	Patients with HF and LVEF ≤35%	Sacubitril-valsartan 100 mg twice daily for 2 wk followed by 200 mg twice daily (condensed regimen) vs 50 mg twice daily for 2 wk and 100 mg twice daily for 3 wk, followed by 200 mg twice daily (conservative regimen)	76% of the patients achieved and maintained sacubitril/valsartan 200 mg twice daily without dose interruption/down-titration over 12 wk (77.8% vs 84.3% for condensed vs conservative; *P =* 0.078)
EVALUATE-HF^67^ 2019	HFrEF	≥50 years of age History of hypertension Chronic HF with LVEF ≤40% NYHA functional class I-III	Sacubitril-valsartan vs enalapril.	No statistically significant difference at 12 weeks between groups in the change of aortic stiffness from baseline
PIONEER-HF^58^ 2019	ADHF	Patients with primary diagnosis of ADHF LVEF ≤40% NT-proBNP ≥1,600 pg/mL or BNP >400 pg/mL	Sacubitril-valsartan vs enalapril	Significant reduction in NT-proBNP in sacubitril-valsartan group compared with enalapril (46.7% vs -25.3%; ratio of change: 0.71; 95% CI: 0.63-0.81; *P <* 0.001)
TRANSITION^62^ 2019	ADHF	Patients hospitalized for ADHF with NYHA functional class II–IV, SBP ≥100 mm Hg, and LVEF ≤40%	Open-label LCZ696 sacubitril-valsartan	Comparable proportions of patients in the pre- and postdischarge initiation groups achieved the target dose of 97/103 mg twice daily at wk 10
PARAGON^60^ 2019	HFpEF	Patients with NYHA functional class II to IV HF, LVEF ≥45%, elevated level of natriuretic peptides, and structural heart disease	Sacubitril valsartan vs valsartan	Sacubitril-valsartan did not result in a significantly lower rate of total hospitalizations for HF and cardiovascular death (rate ratio: 0.87; 95% CI: 0.75-1.01)
PROVE-HF^66^ 2019	HFrEF	Patients with HFrEF who are candidates for on-label sacubitril/valsartan treatment per the standard of care with NYHA functional class II-IV HF and LVEF ≤40%	Open-label sacubitril-valsartan	Reduction in NT-proBNP concentration was weakly yet significantly correlated with improvements in markers of cardiac volume and function at 12 months
PARALLAX^68^ 2021	HFpEF	Patients with HF and LVEF >40%	Sacubitril-valsartan vs enalapril, valsartan, or placebo stratified by prior use of a RAS inhibitor	Sacubitril/valsartan treatment compared with standard RAS inhibitor treatment or placebo resulted in a significantly greater decrease in plasma NT-proBNP levels at 12 wk but did not significantly improve 6-min walk distance at 24 wk
PARADISE-MI^61^ 2021	Post-MI	Patient with MI and evidence of LV systolic dysfunction and/or pulmonary congestion requiring IV treatment and at least 1 of the following 8 risk factors: Age ≥70 y eGFR <60 mL/min/1.73 m^2^ Diabetes mellitus History of prior MI Atrial fibrillation LVEF <30% Worst Killip class III or IV STEMI without reperfusion	Sacubitril-valsartan vs ramipril	Time to first CV death, HF hospitalization, or outpatient HF not different between sacubitril-valsartan vs ramipril The primary outcome occurred in 11.9% of the sacubitril-valsartan group and 13.2% of the ramipril group (HR: 0.90; 95% CI: 0.78-1.04; *P =* 0.17).
LIFE^59^ 2022	Advanced HFrEF	Advanced HFrEF LVEF ≤35% NYHA functional class IV Or patients who require chronic inotropic therapy	Sacubitril-valsartan vs valsartan	Changes in NT-proBNP were not different between sacubitril/valsartan and valsartan The estimated ratio of change in the NT-proBNP AUC of sacubitril-valsartan to valsartan groups was 0.95 (95% CI: 0.84-1.08; *P =* 0.45)

ADHF = acute decompensated heart failure; AUC = area under the curve; BNP = B-type natriuretic peptide; CV, cardiovascular; EVALUATE-HF = Study of Effects of Sacubitril/Valsartan vs Enalapril on Aortic Stiffness in Patients With Mild to Moderate HF With Reduced Ejection Fraction; HF = heart failure; HFpEF = heart failure with preserved ejection fraction; HFrEF = heart failure with reduced ejection fraction; LIFE = LCZ696 in Advanced HF; LVEF = left ventricular ejection fraction; MI = myocardial infarction; NT-proBNP = plasma N-terminal pro–B-type natriuretic peptide; NYHA = New York Heart Association; PARADIGM-HF = Prospective Comparison of ARNi With ACE Inhibitors to Determine Impact on Global Mortality and Morbidity in HF Trial; PARADISE-MI = Prospective ARNi vs ACE Inhibitor Trial to Determine Superiority in Reducing HF Events After MI; PARAGON = Prospective Comparison of ARNi With ARB Global Outcomes in HF With Preserved Ejection Fraction; PARALLAX = Prospective Comparison of ARNi vs Comorbidity-Associated Conventional Therapy on Quality of Life and Exercise Capacity; PARAMOUNT = Prospective Comparison of ARNi with ARB on Management of HF With Preserved Ejection Fraction; PIONEER-HF = Comparison of Sacubitril-Valsartan vs Enalapril on Effect of NT-proBNP in Patients Stabilized From an Acute HF Episode; PROVE-HF = Prospective Study of Biomarkers, Symptom Improvement, and Ventricular Remodeling During Sacubitril/Valsartan Therapy for HF; RAS, renin angiotensin system; SBP = systolic blood pressure; STEMI = ST-segment elevation myocardial infarction; TITRATION = Safety and Tolerability of Initiating LCZ696 in Heart Failure Patients; TRANSITION = Comparison of Pre- and Postdischarge Initiation of LCZ696 Therapy in HFrEF Patients After an Acute Decompensation Event; 6MWT = 6-minute walk test.

### Studies in patients with HFrEF

PARADIGM-HF^11^ (Prospective Comparison of ARN Inhibitors With ACE Inhibitors to Determine Impact on Global Mortality and Morbidity in HF Trial) was a paradigm-changing trial. The trial was stopped early because of the significant reduction in cardiovascular death or HF hospitalization by 20% with sacubitril and valsartan compared with enalapril alone (Table 2). Sacubitril-valsartan was also associated with a significant reduction in HF hospitalization rates and with improvement in both symptoms and physical limitations of HF.^11^ Results of the PARADIGM trial led to the incorporation of ARN inhibitors as a recommendation for treatment of patients with HFrEF in guidelines.^63^

### Studies in patients with acute HF

The PIONEER-HF^58^ (Comparison of Sacubitril-Valsartan vs Enalapril on Effect of N-Terminal Pro–Brain Natriuretic Peptide [NT-proBNP] in Patients Stabilized From an Acute HF Episode) trial further validated the benefits and safety of initiating sacubitril-valsartan in patients hospitalized for acute decompensated HF. In this trial, sacubitril-valsartan was superior to enalapril to reduce NT-proBNP levels in patients with HFrEF (Table 2). The subsequent open-label TRANSITION (Comparison of Pre- and Postdischarge Initiation of LCZ696 Therapy in HFrEF Patients After an Acute Decompensation Event) trial demonstrated that a strategy of sacubitril-valsartan initiation before discharge or shortly after discharge was feasible and safe in patients stabilized after hospitalization for HFrEF.^64^ PARAGLIDE-HF (Changes in NT-proBNP and Outcomes, Safety, and Tolerability in HFpEF Patients With Acute Decompensated Heart Failure Who Have Been Stabilized During Hospitalization and Initiated In-Hospital or Within 30 Days Postdischarge; NCT03988634) is an ongoing study that will address the effect of sacubitril-valsartan compared with valsartan on time-averaged proportional change in NT-proBNP over 8 weeks among patients with HFpEF.

### Studies in patients with HFpEF

Although these trials established the benefit of ARN inhibitors in patients with HFrEF, several studies investigated the safety and efficacy of ARN inhibitors in patients with HFpEF. In the PARAMOUNT (Prospective Comparison of ARN Inhibitors with ARB on Management of HF With Preserved Ejection Fraction) phase II trial, NT-proBNP was significantly reduced at 12 weeks with LCZ696 (sacubitril-valsartan) treatment compared with valsartan.^65^

In the subsequent phase III study, the PARAGON-HF^60^ (Prospective Comparison of ARN Inhibitors With ARB Global Outcomes in HF With Preserved Ejection Fraction) double-blinded trial in patients with New York Heart Association (NYHA) functional class II to IV HF with a left ventricular ejection fraction (LVEF) of ≥45%, sacubitril-valsartan showed no significant difference compared to valsartan in reducing the primary endpoints of death from cardiovascular causes and hospitalizations for HF (Table 2). Subgroup analysis showed benefit with sacubitril-valsartan among those with an ejection fraction (EF) in the lower range (EF: <57%) and women.^60^

### Studies addressing secondary endpoints, reverse remodeling, aortic stiffness, biomarkers, exercise capacity, and quality of life

In PROVE-HF (Prospective Study of Biomarkers, Symptom Improvement, and Ventricular Remodeling During Sacubitril/Valsartan Therapy for HF), a prospective, single-group, open-label exploratory study of patients with HFrEF treated with sacubitril-valsartan,^66^ reduction in NT-proBNP concentration was weakly yet significantly correlated with improvements in markers of cardiac volume and function. At 12 months, LVEF increased and left ventricular (LV) end-diastolic and end-systolic volumes decreased significantly, suggestive of reverse cardiac remodeling with ARN inhibition.^66^ In the randomized, double-blind EVALUATE-HF (Study of Effects of Sacubitril/Valsartan vs Enalapril on Aortic Stiffness in Patients With Mild to Moderate HF With Reduced Ejection Fraction), sacubitril-valsartan, compared with enalapril, did not significantly reduce the primary endpoint of central aortic stiffness or the prespecified secondary endpoint of LVEF.^67^ In the PARALLAX (Prospective Comparison of ARN Inhibition vs Comorbidity-Associated Conventional Therapy on Quality of Life and Exercise Capacity) trial, in patients with HF with an LVEF of >40%, sacubitril-valsartan resulted in a greater reduction in NT-proBNP levels than for those in the comparator group (Table 2).^68^ At week 24, there was no significant difference in the 6-minute walk distance, Kansas City Cardiomyopathy Questionnaire clinical summary score, or improvement in NYHA functional class.^68^

### Studies in the pediatric population

The efficacy of sacubitril-valsartan in comparison with enalapril is being evaluated in a multinational, randomized, double-blind trial in pediatric patients with HF (NYHA/Ross class II-IV) and systemic LV systolic dysfunction (LVEF ≤40%), PANORAMA-HF (Study to Evaluate Safety, Tolerability, Pharmacokinetics and Pharmacodynamics of LCZ696 in Pediatric Patients With HF).^69^ Based on an early analysis of 110 pediatric patients, the reduction in NT-proBNP over 12 weeks was 44% and 33% in the sacubitril-valsartan and enalapril groups, respectively, but it did not reach statistical significance. The reductions in NT-proBNP with sacubitril-valsartan were similar to or larger than what was seen in adults and were considered a reasonable basis from which to infer improved cardiovascular outcomes in children, resulting in FDA approval for sacubitril-valsartan in the pediatric population.^70^

### Studies in patients post-MI

The safety and efficacy of ARN inhibitors in patients following MI was investigated in the PARADISE-MI (Prospective ARN Inhibitor vs ACE Inhibitor Trial to Determine Superiority in Reducing HF Events After MI).^61^ Patients were randomized within 7 days after acute MI to receive sacubitril-valsartan or ramipril. Patients were required to have no prior diagnosis of HF but have either transient pulmonary congestion or an EF of ≤40% and at least 1 other factor that increased their risk for HF or death (Table 2). Compared to ramipril, sacubitril-valsartan was not associated with an improvement in clinical endpoints (Table 2). The trial had sufficient power to detect the treatment effect size anticipated, but it was also noted that the mortality rates were significantly lower than historical MI trials with ACE inhibitors. The drug initiation differed from the PARADIGM trial because treatment with either an ACE inhibitors or ARNi was initiated without a run-in phase.^67^

### Studies in patients with advanced HF

PARADIGM-HF established the benefit of ARNi therapy over ACE inhibitors for patients with HFrEF; however, the study population included predominantly patients with NYHA functional class II to III symptoms. Less than 1% of the study population had NYHA functional class IV symptoms.^11^ Because of limited clinical evidence in patients with NYHA functional class IV, the use of sacubitril-valsartan was recommended only in patients with NYHA functional class II to III HF by practice guidelines in 2017.^63^ Subsequently, the LIFE (LCZ696 in Advanced HF) trial was designed as a 24-week randomized, double-blinded control trial to assess the tolerability, efficacy, and safety of sacubitril-valsartan compared with valsartan in patients with advanced HFrEF (LVEF ≤35%) and recent NYHA functional class IV symptoms.^59^ Patients underwent an unblinded run-in period with sacubitril-valsartan. There were no differences between the 2 treatment groups regarding the primary endpoint of the area under the curve for the ratio of NT-proBNP compared with baseline. Interestingly, the secondary efficacy endpoint of the number of patient-days alive, out of hospital, and without HF events was numerically higher (ie, better) in the valsartan arm (median: 157.0 days; IQR: 53.5-164.0 days) compared with the sacubitril-valsartan arm (median: 147.0 days; IQR: 9.0-164.0 days), but this did not reach statistical significance. The HR for cardiovascular death or first HF hospitalization was 1.32 (95% CI: 0.86-2.03; *P =* 0.20) and for HF hospitalizations was 1.24 (95% CI: 0.80-1.93; *P =* 0.33) for sacubitril-valsartan compared to valsartan. The estimated difference between the 2 groups was -11.2 days (95% CI: -26.4 to 4.0; *P* = 0.15). Because neprilysin inhibition was expected to improve HF outcomes, the results of the LIFE trial were surprising.^11^ The study was not powered to examine clinical endpoints because of its small sample size, the relatively short duration, and COVID-19 mitigation strategies that affected the enrollment. The decrease in the number of randomized patients from the originally planned 400 to 335 nominally reduced the statistical power to detect a 20% treatment difference from 88% to 79%.^59^

## Unanswered Questions

### Effects of neprilysin inhibition in certain phenotypes of HF patients

#### Patients with NYHA functional class IV symptoms

Despite statistical insignificance, the numerically higher event rates in the sacubitril-valsartan arm in the LIFE trial raised the possibility of a lack of efficacy with neprilysin inhibition in advanced HF patients with NYHA functional class IV symptoms.^11^ Although the LIFE trial was not powered to examine clinical endpoints, in the much larger PARADIGM trial, there was a similar signal.^11^ By subgroup analysis, although there was a reduction in cardiovascular death or HF hospitalization rates with sacubitril-valsartan compared with enalapril in patients with NYHA functional class I to II symptoms, there was no benefit in patients with NYHA functional class III to IV HF.^11^ The interaction between NYHA functional class and the primary endpoint was significant for heterogeneity *(P =* 0.03).^11^ In PARADIGM, approximately 24% of patients had NYHA functional class III and 0.7% had NYHA functional class IV symptoms at baseline. This raises the question of whether neprilysin inhibition is ineffective in advanced HF patients with NYHA functional class IV symptoms.

The following observations may provide some explanations for these findings and highlight the need for further research. In advanced HF, there is diminished responsiveness to natriuretic peptides in target organs despite dramatic increases in circulating natriuretic peptide concentrations.^71^ Among patients with NYHA functional class III to IV HF, despite high levels of plasma natriuretic peptides, the plasma cGMP levels do not rise and reach a plateau, suggesting down-regulation of natriuretic peptide receptors coupled to guanylate cyclase.^72^ Among patients with mild HF, plasma cGMP levels correlate with ANP levels; in contrast, these correlations are usually not found in patients with moderate to severe HF.^72^ There is an inverse relationship between plasma BNP levels and circulating neprilysin activity.^73^ Notably, in a study of patients with acute decompensated heart failure, patients with elevated BNP levels over 916 pg/mL exhibited an almost 3-fold reduction in circulating neprilysin activity compared with those with lower BNP levels.^73^ Although neprilysin concentrations were moderately higher in patients with elevated BNP levels, the neprilysin activity was markedly lower. These findings suggest that BNP-mediated neprilysin inhibition may occur when BNP rises above a critical threshold, and elevated BNP may act as a “molecular switch” that participates in the accumulation of bioactive vasoactive peptides by the inhibition of neprilysin. This raises the question of whether neprilysin inhibition may be the most effective in patients with mild to moderate HF, when neprilysin activity is high, but not in patients with very advanced HF with markedly elevated BNP and neprilysin concentrations but low neprilysin activity.^73^ There is also an attenuated renal response to natriuretic peptides in patients with HF.^74^^,^^75^ Urine volume and sodium excretion fail to increase, and the rises in plasma and urinary cGMP levels are diminished in patients with HF compared to healthy control individuals.^75^ Furthermore, vasodilatory responses to natriuretic peptides are diminished in HF patients,^76^^,^^77^ and there is significant down-regulation in the density of natriuretic peptide receptors in the myocardium and smooth muscles.^78^ This is coupled with significant desensitization of natriuretic peptide receptors, which results in the inability to crosslink ligand and bind the hormone.^79^ Exogenous administration of natriuretic peptides fails to lower the plasma renin activity and plasma aldosterone or noradrenaline concentrations in patients with HF.^74^^,^^80^^,^^81^ In experimental models of HF, sodium excretion, renal blood flow, renal vascular reactivity, and urinary ANP and cGMP excretion in response to neprilysin inhibition are markedly lower in severe HF compared to mild HF or control individuals.^82^ Furthermore, there is evolving evidence that suggests BNP circulates in different structural forms that affect HF in vivo activity. Despite the high levels reported by conventional assays, there is evidence of the absence of certain active subcomponents of BNP by immunoaffinity purification assays in advanced HF patients, suggesting the existence of altered forms of BNP in severe HF that may be detected by conventional assays but may not be present or functionally active.^83^ These may explain, in part, the attenuated natriuretic peptide responses in patients with advanced HF. Therefore, the prevention of enzymatic breakdown of natriuretic peptides may not be a very effective solution in advanced HF patients when there is downstream blunting of the response.

Furthermore, in patients with advanced HF with NYHA functional class IV symptoms, there is a possibility that neprilysin inhibition may result in the potentiation of vasoconstrictive peptides such as angiotensin I and II and endothelin. RAAS activation may override the effect of natriuretic peptides and further impair natriuretic peptide responsiveness. In experimental models, there is evidence of suppressed vascular, hormonal, and renal responses to natriuretic peptides after angiotensin II^84^ or endothelin 1 infusion.^85^ ANP-induced accumulation of cGMP is significantly inhibited in the presence of angiotensin II,^86^ and elevated angiotensin levels may lead to natriuretic peptide receptor down-regulation.^71^ Future studies are needed to provide mechanistic insights—eg, whether plasma or urinary cGMP coupled with ANP levels could be an important surrogate for the prediction of response to ARNi therapy among patients with advanced HF.

Although there are reports of consistent benefit with sacubitril-valsartan across different risk groups of HF patients in the PARADIGM-HF trial assessed by the MAGGIC (Meta-analysis Global Group in Chronic HF) and EMPHASIS-HF (Eplerenone in Mild Patients Hospitalization and Study in HF) risk scores,^87^^,^^88^ these scores do not help characterize a specific advanced HF phenotype or neurohormonal profiles.^87^ They incorporate a wide range of variables related to future risk at the population level but not at the individual patient level.^89^ For example, the EMPHASIS-HF score does not take into account the NYHA functional class. (All patients in EMPHASIS-HF were in NYHA functional class II.)^88^ Therefore, consistent benefit across such different risk groups would not be adequate to validate benefit in advanced HF with NYHA functional class IV symptoms. Similarly, in the PARADIGM-HF trial, patients deemed to be most clinically stable by virtue of never having had a prior HF hospitalization or having had only a remote HF hospitalization before randomization in PARADIGM-HF benefited at least as much from sacubitril-valsartan therapy as less stable patients with a recent history of hospitalization,^90^ but these analyses did not explore efficacy and safety in advanced HF patients with NYHA functional class IV symptoms or in patients with repeated HF hospitalizations.

cGMP is degraded by cellular phosphodiesterases (PDEs), such as PDE5 or PDE9.^91^ Enhanced PDE activity in HF may contribute to reduced and blunted response to natriuretic peptides in HF by impairing its intracellular signal transduction pathways.^91^^,^^92^ In a study in dogs with tachypacing-induced HF, acute administration of a selective PDE5 inhibitor achieved similar hemodynamic responses to treatment with exogenous BNP and exerted an additive effect to BNP administration.^92^ The reduced ratio of plasma cGMP to plasma BNP seen in HF was ameliorated by PDE5 inhibition but had no effect in nonfailing animals. Natriuretic peptide desensitization in HF may relate, in part, to increased PDE activity, supporting a therapeutic role for PDE5 or PDE9 inhibition, especially among patients with advanced HF with blunted downstream response to natriuretic peptides.^91^^,^^92^ Whether this approach can be effective in patients with advanced HF awaits further studies.

#### Patients post-MI

In the PARADISE-MI trial, sacubitril-valsartan was not associated with an improvement in clinical endpoints compared with ramipril in patients with high-risk features following acute MI.^61^ These findings argue against the use of ARNi a short time after an acute MI^93^ and raise questions about whether neprilysin inhibition adds any benefit post-MI. Enhanced natriuretic peptide degradation and elevated cardiac neprilysin activity have been shown in HF patients but not in post-MI patients.^17^ In a clinical study of acute MI patients, neprilysin levels did not change significantly in the first hours or 1-month period following reperfusion in ST-segment elevation myocardial infarction patients. There was no significant relationship between circulating neprilysin levels with markers of infarct size, troponin, and inflammation or with 1-year adverse outcomes.^94^

In an earlier prospective, multicenter, randomized, double-blind, active-comparator trial in patients with asymptomatic LV systolic dysfunction late after MI, treatment with sacubitril-valsartan compared with valsartan did not significantly reduce LV end-systolic or end-diastolic volume indices.^95^ There were no significant between-group differences in NT-proBNP, high-sensitivity cardiac troponin, left atrial volume index, LVEF, LV mass index, or patient global assessment of change.^95^

Experimental evidence also does not provide any justification for neprilysin inhibition in MI in the absence of chronic HF. In a pig model of MI, plasma neprilysin levels did not change after acute MI in the first hours or in 3 weeks.^96^ In other experimental animal models of MI, although neprilysin inhibition with omapatrilat prevented degradation of bradykinin,^97^ it did not result in increased survival or other beneficial results.^98^ In another experimental model of HF following MI in rats, sacubitril-valsartan attenuated progressive LV dilation, improved global LV function, limited remodeling in the remote and border zones, and increased perfusion to the infarct after 5 weeks of treatment.^99^ However, this was an experimental model of HF following MI and not MI alone. These results suggest that in the setting of acute MI, in the absence of development of HF, there is no evidence of increased neprilysin activity or enhanced natriuretic peptide degradation to warrant neprilysin inhibition. This, at least in part, may explain the lack of improvement with sacubitril-valsartan in the PARADISE-MI trial.^61^

### Metabolic effects

Emerging evidence suggests that neprilysin hydrolyzes peptides that play an important role in glucose metabolism, such as glucagon-like peptide-1.^100^ Inhibition of the degradation of this peptide can result in an improvement in blood glucose levels.^101^ Neprilysin activity is increased in obesity and correlates with decreased insulin sensitivity and reduced beta-cell function.^101^ In PARADIGM-HF, treatment with sacubitril-valsartan resulted in a greater reduction in glycated hemoglobin than treatment with enalapril in patients with preexisting diabetes mellitus.^102^ The initiation of insulin or oral glucose-lowering medications was also lower in the sacubitril-valsartan group.^102^

A couple of important orexigenic and anorexigenic compounds are also known substrates for hydrolysis by neprilysin. In neprilysin knockout mice, there was evidence of late onset excessive gain in body weight with a normocaloric diet exclusively from the accumulation of fat tissue accompanied by a deregulation of lipid metabolism, higher blood glucose levels, and impaired glucose tolerance.^34^ In that study, a lack of neprilysin activity, genetically or pharmacologically, led to a gain in body fat.^34^ To date, there have not been any clinical studies demonstrating adverse metabolic effects, weight gain, or obesity with neprilysin inhibition in patients, including those with HF. The experimental findings underline the need for long-term studies to determine the metabolic effects of neprilysin inhibition on weight, obesity, glycemic control, and lipid profile.

### Proteinuria and glomerular filtration effects

The effects of natriuretic peptides on the kidney are not unidirectionally favorable. Natriuretic peptides have been shown to contribute to the pathogenesis of glomerular hyperfiltration in diabetes in experimental animal and human studies.^103^, ^104^, ^105^ Infusion of ANP increases the urinary excretion of albumin in patients with diabetes.^104^^,^^105^ Increased albuminuria is attributed to a rise in glomerular pressure but might at least partly result from an attenuation of tubular protein reabsorption.^104^ More prominent effects were also reported in patients with nondiabetic renal disease and nephrotic syndrome.^105^

Interestingly, in experimental studies, ARNi resulted in favorable effects on diabetic nephropathy,^106^^,^^107^ accompanied by improvements in RAAS profile and inhibition inflammation, fibrosis, and apoptosis.^106^, ^107^, ^108^ In PARADIGM-HF, compared with patients treated with enalapril, those treated with sacubitril-valsartan had a slower rate of decline in estimated glomerular filtration rate (eGFR), and the magnitude of the benefit was larger in patients with vs those without diabetes.^109^ However, there was also a greater increase in the urinary albumin/creatinine ratio (UACR) with sacubitril-valsartan when compared to enalapril.^110^ In PARADIGM-HF, 24% of the patients had an increased UACR. The effect of sacubitril-valsartan on cardiovascular death or HF hospitalization was not modified by eGFR or increase in UACR.^110^ Similarly, in the PARAMOUNT trial of patients with HFpEF, the eGFR declined less in the LCZ696 group than in the valsartan group, but over 36 weeks, the geometric mean of UACR increased in the LCZ696 group.^111^ Also, in the PARALLAX trial of patients with HFpEF with an LVEF of >40%, the risk of albuminuria was higher with sacubitril-valsartan when compared with standard renin-angiotensin system inhibitor treatment (12.3% vs 7.6%).^68^

Potential explanations for these observations of an increase in albuminuria despite slowing of the decline in eGFR with neprilysin inhibitors or ARNi include the following. Enhanced renal bioavailability of natriuretic peptides in addition to reduction in systemic BP and renal perfusion pressure may result in a preferential vasorelaxation of the afferent arteriole and a relative vasoconstriction of the efferent arteriole.^105^ This can contribute to increasing intracapillary hydraulic pressure despite a decreased renal perfusion pressure, which can subsequently increase the filtration fraction and preserve GFR in a reduced BP setting.^105^^,^^112^ These may amplify a defect in the size selectivity of the glomerular barrier with a secondary increase in the filtering surface area, resulting in increased vascular permeability. The increased intracapillary hydraulic pressure combined with a direct effect of natriuretic peptides may increase albumin ultrafiltration and result in an increase in albuminuria.^105^^,^^112^^,^^113^ This hypothesis needs confirmation in future studies measuring kidney perfusion and filtration and long-term kidney function outcomes.^105^

A meta-analysis of 3 trials in HFrEF that compared combined neprilysin with RAAS inhibition with RAAS inhibition alone (IMPRESS [comparison of vasopeptidase inhibitor, omapatrilat, and lisinopril on exercise tolerance and morbidity in patients with heart failure]: omapatrilat vs lisinopril; OVERTURE: omapatrilat vs enalapril; and PARADIGM-HF: sacubitril-valsartan vs enalapril) demonstrated that combined neprilysin/RAAS inhibition was associated with a reduced incidence of a rise in serum creatinine and a less pronounced decline of GFR despite more hypotension.^114^ The UK HARP-III (United Kingdom Heart and Renal Protection III) trial demonstrated that sacubitril-valsartan had similar effects on kidney function and albuminuria as irbesartan over 12 months, but it had the additional effect of lowering BP and cardiac biomarkers in people with chronic kidney disease.^115^

Albuminuria is an independent factor for renal and cardiovascular risk and an independent predictor of prognosis in HF.^116^ Whether the increase in urinary albumin with ARNi will translate into an excess risk of renal events in subjects with HF needs further exploration in longer-term trials.^105^ Natriuretic peptide-induced impairment of tubular handling of other ultrafiltered proteins such as such as β2-microglobulin and free κ-light chains may also need to be also be taken into consideration.^104^^,^^105^ Longer-term studies evaluating glomerular perfusion, filtration, and the permeability of proteins implicated in cardiovascular health and their association with clinical endpoints will provide greater insights into the mechanisms of actions with these agents.

### Comparator group in studies with ARNi

Another area of discussion is the active treatment comparator in randomized clinical trials with ARNi. In the LIFE trial of patients with HFrEF and NYHA functional class IV symptoms, the active comparator was valsartan.^59^ Although valsartan was shown to reduce a combined endpoint of mortality and morbidity in the The Valsartan Heart Failure Trial, it did not reduce overall mortality,^117^ and only 1.7% of the VAL-HeFT trial population was in NYHA functional class IV. Subgroup analysis demonstrated benefit for the combined endpoint among patients with NYHA functional class III to IV HF symptoms.^117^ ACE inhibitors, specifically enalapril, on the other hand, were shown to significantly reduce mortality from the progression of HF among patients with NYHA functional class IV HF symptoms in CONSENSUS (Cooperative North Scandinavian Enalapril Survival Study).^118^ Although CONSENSUS was conducted more than 3 decades ago and does not represent current therapy, the magnitude of risk reduction for mortality with enalapril (approximately 50% in NYHA functional class IV patients) raises the question pf whether enalapril would have been a better comparator and may have achieved significance and superiority in the reduction of clinical endpoints when compared against sacubitril-valsartan in the LIFE trial. It is important for the comparator arm to reflect the best evidence-based treatment for the targeted population.

### Hypotension and tolerability

In most of the clinical studies with ARNi, a run-in period was used to ascertain tolerability. In PARADIGM-HF, approximately 20% of participants discontinued the study drug during the run-in phase (10.4% during enalapril run-in; 10.3% during sacubitril-valsartan run-in) because of intolerance or for other reasons.^11^ Patients with higher natriuretic peptide levels, lower BP, lower GFR, and more severe HF were at higher risk for noncompletion during the run-in period.^119^ Among patients who completed the run-in period and were randomized, symptomatic hypotension occurred more frequently in the sacubitril-valsartan group than in those receiving enalapril (14.0% vs 9.2%; *P <* 0.001).^11^ Hypotension was more likely to occur in older patients, those with a lower systolic BP at screening, and those taking doses lower than the target doses of ACE inhibitors/ARBs before enrollment.^120^ Interestingly, patients with a hypotensive episode during run-in but who ultimately could be randomized derived similar benefit from sacubitril-valsartan compared with enalapril as those who did not experience hypotension.^120^

In PIONEER, which included patients with HFrEF who were hospitalized for acute decompensated HF, patients were required to be hemodynamically stable with a systolic BP of at least 100 mm Hg. Rates of symptomatic hypotension did not differ significantly between the sacubitril-valsartan and enalapril groups. In PARAGON-HF with patients with HFpEF,^60^ sacubitril-valsartan was also associated with a higher rate of hypotension compared with enalapril (15.8% vs 10.8%; *P <* 0.001). This trial excluded patients with a systolic BP of <110 mm Hg at the first visit or a systolic BP of <100 mm Hg or symptomatic hypotension in other visits. During a single-blind run-in period, 16% of patients discontinued the study drug.^60^ In the PARALLAX trial, which included patients with HFpEF with an LVEF of >40%, sacubitril-valsartan was associated with higher rates of hypotension compared with control groups (14.1% vs 5.5%), especially in the stratum with no renin-angiotensin system inhibitor.^68^ In PARADISE-MI, which did not have a run-in period, sacubitril-valsartan was again associated with a higher rate of hypotension compared with ramipril (28% vs 22%; *P <* 0.001).^61^ In an open-label exploratory study of patients with HFrEF, PROVE-HF, treatment with sacubitril-valsartan over 12 months was associated with hypotension and dizziness, which were noted in 17.6% and 16.8% of patients, respectively.^66^

In the LIFE trial, which was a smaller trial that enrolled patients with HFrEF with NYHA functional class IV HF symptoms, although the rates of hypotension in the sacubitril-valsartan arm did not reach significance when compared with the valsartan arm (17% vs 12%; *P =* 0.16), 18% of patients were not able to tolerate a lower dose of sacubitril-valsartan (100 mg/d) during the short run-in period, and 29% discontinued sacubitril-valsartan during the 24 weeks of the trial.^59^ Less than 35% of the patients were receiving the target dose of 400 mg/d of sacubitril-valsartan at the end of the study.^59^ The authors acknowledged that the safety and tolerability of sacubitril-valsartan may have been different than observed if the patients had not undergone a run-in phase with low-dose sacubitril-valsartan.^59^

These findings underscore the recognition of BP-lowering effects of ARNi and its effect on tolerability. In studies with a run-in phase for tolerability, the rates of hypotension were 14% to 16%,^120^ whereas in studies without a run-in period, the rate of hypotension was as high as 28%.^61^ It should be kept in mind that neprilysin knockout mice have significantly lower BP and are prone to shock.^21^ Because most studies with ARNi excluded patients with hypotension or a systolic BP of >100 mm Hg, ARNi is not recommended in patients with hypotension, and the safety of ARNi in patients with hypoperfusion or shock is not known.^114^

### Amyloid deposition

Because neprilysin is partially responsible for the degradation of Aβ, the peptide implicated in Alzheimer dementia, there is a theoretical concern about the long-term effects of sacubitril-valsartan on cognition. Sacubitril and valsartan are highly bound to plasma proteins (94%-97%), and sacubitril is thought to cross the blood-brain barrier to a limited extent (0.28%).^70^ The effects of sacubitril-valsartan on Aβ concentrations in CSF and brain tissue were assessed in young cynomolgus monkeys treated with sacubitril-valsartan for 2 weeks.^121^ Despite low CSF and brain penetration, CSF exposure to sacubitril was sufficient to inhibit neprilysin and resulted in an increase in the CSF levels of Aβ 1-40, Aβ 1-42, and total Aβ.^121^ However, there were no elevations in any Aβ isoforms in the brains of these monkeys on day 16. In a second study—a toxicology study—cynomolgus monkeys were administered sacubitril-valsartan (300 mg/kg) for 39 weeks; no microscopic brain changes or Aβ deposition were present by immunohistochemical staining.^121^

In healthy volunteers, administration of sacubitril-valsartan (400 mg) once daily for 2 weeks was associated with an increase in CSF Aβ 1-38 levels compared to placebo, but there were no changes in concentrations of CSF Aβ 1-40 or CSF Aβ 1-42.^122^ The clinical relevance of this finding is unknown. There was no evidence that sacubitril-valsartan, compared with enalapril, increased dementia-related adverse events in PARADIGM-HF, although longer follow-up may be necessary to detect such a signal with sensitive tools to detect lesser degrees of cognitive impairment.^123^ The rates of dementia-related adverse events in both treatment groups in PARADIGM-HF were similar to those in 3 other recent trials in HFrEF.^123^ In an analysis of adverse event cases submitted to the FDA Adverse Event Report System from July 2015 to March 2017, cognition- and dementia-related adverse events associated with sacubitril-valsartan (5.1%) were lower than the proportion of these reports with other medications (6.6%; reporting OR: 0.72; 95% CI: 0.65-0.79). Restricting the comparison to cases with age >60 years and with the use of a comparator group with HF resulted in no association between sacubitril-valsartan and dementia-related adverse events.^124^

The ongoing PERSPECTIVE (Efficacy and Safety of LCZ696 Compared to Valsartan on Cognitive Function in Patients With Chronic HF and Preserved Ejection Fraction [NCT02884206]) trial is assessing the long-term neurocognitive effects and safety of sacubitril-valsartan compared with valsartan. This study uses a battery of validated neurocognitive instruments and advanced imaging for amyloid deposition in more than 550 patients with HFpEF.

Deposition of Aβ in the retina is known to contribute to the development of age-related macular degeneration.^32^ Neprilysin-deficient mice develop retinal degeneration and subretinal deposits similar to age-related macular degeneration.^32^ Intravitreal administration of neprilysin decreased ocular Aβ levels.^33^ In clinical trials with ARNi, there were no increased events of vision loss, but systematic screening and long-term follow-up were not performed to monitor subepithelial retinal Aβ deposits for macular degeneration. A systems biology approach to detect patients who may be prone to macular degeneration with sacubitril-valsartan has been proposed.^125^

Individuals with a genetic predisposition to Alzheimer disease or macular degeneration may be at a higher risk for adverse effects of neprilysin inhibition and Aβ deposition. Polymorphisms in the neprilysin gene with loss of function have been associated with increased susceptibility to Alzheimer disease.^29^^,^^30^ Pharmacogenomics can potentially explain the variability in the effect of the ARNi and its side effects. In the future, genetic testing and genomic testing for neprilysin polymorphisms may play an important role in monitoring for long-term side effects in ARNi-treated HF patients.^126^ Whether a rise in plasma or CSF Aβ would be a risk for the future development of Alzheimer dementia or macular degeneration can also be explored. Longer-term studies are needed to determine long-term effects of ARNi on cognitive function and macular degeneration.

## Conclusions

As demonstrated in this review, the benefits of neprilysin inhibition are dependent on specific patient diagnoses and patient characteristics. The evidence supporting ARNi use over ACE inhibitors or ARBs is strongest for patients with a diagnosis of NYHA functional class II to III HFrEF. Patients with advanced HFrEF (NYHA functional class IV) or patients post-MI without HF do not seem to gain much benefit from the addition of a neprilysin inhibitor to their medication regimen. In line with the evidence presented here, the American College of Cardiology/American Heart Association guidelines recommend initiation of ARNi or replacing ACE inhibitors or ARBs with ARNi in patients with HFrEF NYHA functional class II or III but not with NYHA functional class IV symptoms as Class I recommendations.^63^^,^^127^ The European Society of Cardiology guidelines take a more cautious approach and recommend ARNi as a replacement for ACE inhibitors in patients with HFrEF as a Class I recommendation and initiation in ACE inhibitor-naive (ie, de novo) patients with HFrEF as a Class IIb recommendation.^128^ Both the European Society of Cardiology and American College of Cardiology/American Heart Association guidelines expression caution for hypotension as a side effect and recommend against use for patients with a history of angioedema and a 36-hour washout period after ACE inhibitors to reduce the risk of angioedema.^63^^,^^127^^,^^128^ Further studies are needed to support the initiation of ARNi, rather than ACE inhibitors/ARBs, as the first-line therapy in patients with advanced NYHA functional class IV HF or in post-MI patients with LV dysfunction. It should be kept in mind that neprilysin degrades a large number of peptides in a variety of organ systems and that not all of its substrates are beneficial, underlining the need for risk and benefit assessment in different phenotypes with longer studies. Specifically, future longer-term studies are needed with ARNi to address unanswered questions, including efficacy and safety in advanced HF patients with NYHA functional class IV symptoms, patients post-MI with or without LV dysfunction who develop albuminuria, those with hypotension and hypoperfusion, or those with risk for Alzheimer disease and macular degeneration.

## Funding Support and Author Disclosures

Dr Bozkurt has served as a consultant for Bayer and scPharmaceuticals; is on the Clinical Events Committee for the Guide-HF Trial by Abbott Pharmaceuticals;and is on the Data Safety Monitoring Board for Anthem Trial by Liva Nova Pharmaceuticals. Dr Misra has served as a site primary investigator for PIONEER HF, PARAGON-HF, and PARAGLIDE-HF studies. All other authors have reported that they have no relationships relevant to the contents of this paper to disclose.
